# Dietary and genetic influences on hemostasis in a Yup’ik Alaska Native population

**DOI:** 10.1371/journal.pone.0173616

**Published:** 2017-04-04

**Authors:** Nicholas T. Au, Morayma Reyes, Bert B. Boyer, Scarlett E. Hopkins, Jynene Black, Diane O’Brien, Alison E. Fohner, Joe Yracheta, Timothy Thornton, Melissa A. Austin, Wylie Burke, Kenneth E. Thummel, Allan E. Rettie

**Affiliations:** 1 Department of Medicinal Chemistry, University of Washington, Seattle, Washington, United States of America; 2 Department of Laboratory Medicine, University of Washington, Seattle, Washington, United States of America; 3 Center for Alaska Native Health Research, University of Alaska Fairbanks, Fairbanks, Alaska, United States of America; 4 Public Health Genetics, University of Washington, Seattle, Washington, United States of America; 5 Department of Pharmaceutics, University of Washington, Seattle, Washington, United States of America; 6 Department of Biostatistics, University of Washington, Seattle, Washington, United States of America; 7 Department of Epidemiology, University of Washington, Seattle, Washington, United States of America; 8 Department of Medical Ethics, University of Washington, Seattle, Washington, United States of America; Institut d'Investigacions Biomediques de Barcelona, SPAIN

## Abstract

Fish and marine animals are important components of the subsistence diet of Alaska Native people, resulting in a high ω3 PUFA intake. The historical record for circumpolar populations highlights a tendency for facile bleeding, possibly related to ω3 PUFA effects on platelet activation and/or vitamin K-dependent clotting factors. To evaluate these two scenarios in Yup’ik people of southwestern Alaska, we examined the association between dietary ω3 PUFA intake and activities of clotting factor II, V, fibrinogen, PT, INR, PTT, and sP-selectin in 733 study participants, using the nitrogen isotope ratio of red blood cells as a biomarker of ω3 PUFA consumption. sP-selectin alone correlated strongly and inversely with ω3 PUFA consumption. Approximately 36% of study participants exhibited PIVKA-II values above the threshold of 2 ng/ml, indicative of low vitamin K status. To assess genetic influences on vitamin K status, study participants were genotyped for common vitamin K cycle polymorphisms in *VKORC1*, *GGCX* and *CYP4F2*. Only CYP4F2*3 associated significantly with vitamin K status, for both acute (plasma vitamin K) and long-term (PIVKA-II) measures. These findings suggest: (i) a primary association of ω3 PUFAs on platelet activation, as opposed to vitamin K-dependent clotting factor activity, (ii) that reduced CYP4F2 enzyme activity associates with vitamin K status. We conclude that high ω3 PUFA intake promotes an anti-platelet effect and speculate that the high frequency of the CYP4F2*3 allele in Yup’ik people (~45%) evolved in response to a need to conserve body stores of vitamin K due to environmental limitations on its availability.

## Introduction

Interactions between environment (diet) and genotype play an important role in determining an individual’s susceptibility to disease and response to environmental agents, including drugs [1]. For native communities living in the circumpolar north, fish and marine animals are important subsistence foods. Such foods are rich in ω3 polyunsaturated fatty acids (ω3 PUFAs), the high consumption of which has been associated with improved health with respect to several chronic disease states [2–6]. Research into the benefits of a high ω3 PUFA diet was stimulated in large part by the early studies of Dyerberg and Bang in Greenland Inuit [7]. These investigators reported that this population, who consumed very high dietary amounts of ω3 PUFAs, exhibited prolonged bleeding times and decreased platelet aggregation relative to Danish controls. Over the past 50 years high ω3 PUFA intake has been associated with a plethora of biological effects relating to cardiovascular physiology and many studies emphasize their beneficial role in cardiac health [8–10].

**Fig 1:**
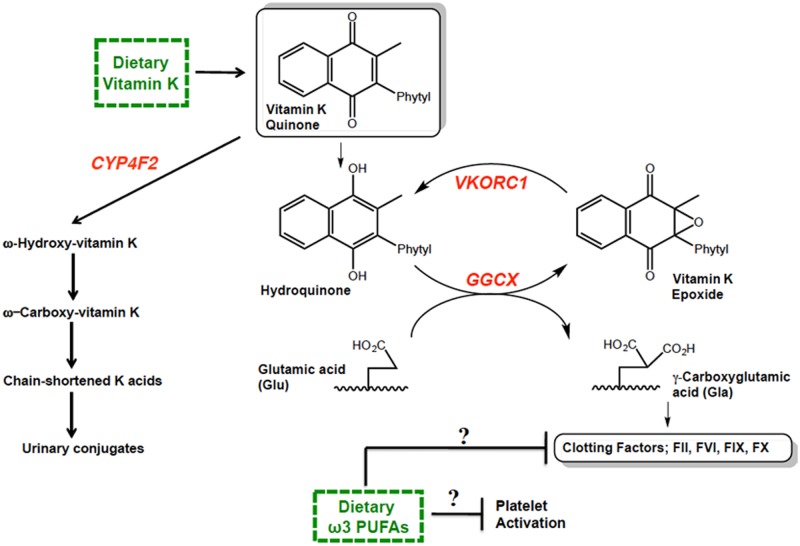
Scheme illustrating potential vitamin K cycle gene-diet interplay in modifying hemostasis. Vitamin K cycle-related genes highlighted in red are *VKORC1*, *GGCX*, *CYP4F2*. The dietary factors investigated; vitamin K and ω3 polyunsaturated fatty acids (ω3 PUFAs), are boxed in green.

A nutritionally-based bleeding diathesis in circumpolar populations might be expected to be modulated by vitamin K status. Vitamin K1 (VK1) has a critical role in coagulation, serving as a cofactor to the enzyme γ-glutamyl carboxylase (GGCX) that catalyzes the posttranslational carboxylation of N-terminal glutamic acid (Glu) residues to γ-carboxy glutamic acids (Gla) on vitamin K-dependent clotting factors (see Fig 1). Some studies conducted in rodents suggest that ω3 PUFAs may precipitate bleeding events through interference with clotting factor activity [11, 12]. However, in humans, the evidence for an effect of ω3 PUFAs on vitamin K-dependent hemostatic measures of coagulation has not been strong [13–15].

It is plausible that circumpolar populations are historically prone to a hypocoagulable state, in part, because of low intake of vitamin K, particularly during seasons when traditional sources such as “tundra greens” and seaweed are unavailable and consumption of commercial greens is limited by access and cost. Recently, we analyzed Alaska Native populations for variation in genes encoding vitamin K recycling (*GGCX*, *VKORC1*) and catabolizing (*CYP4F2*) enzymes (see Fig 1) and found particularly high frequencies in Yup’ik people of variants of *VKORC1* and *CYP4F2* associated with reduced enzyme function [16]. Therefore, in order to better understand how gene-environment interactions might impact the health of Yup’ik people in relation to blood coagulation, we have evaluated the effect of genetic variation in key vitamin K-associated genes on dietary influences in hemostasis. A scheme illustrating potential interplay between these various factors is shown in Fig 1.

This study, therefore, had two main elements. First, we determined the relationship between ω3 PUFA intake and platelet function, clotting factor activity and blood coagulation using the nitrogen isotope ratio (^15^N/^14^N, expressed as the δ^15^N value) in red blood cell (RBC) membranes as a biomarker of dietary ω3 PUFA intake in Yup’ik study participants. This method has been validated as a rapid, medium throughput assay for assigning ω3 PUFA intake status in the Yup’ik population [17]. Importantly, RBCs provide a stable and informative measure of ω3 PUFA intake because they reflect dietary intake over 1–3 months. Second, we measured plasma vitamin K1 and PIVKA-II levels in study participants to assess both acute and longer-term vitamin K status and evaluated associations between these indices of vitamin K status and the common vitamin K cycle polymorphisms; *VKORC1 1173 A>G*, CYP4F2*3 and *GGCX R325Q*. These studies provide new insights into dietary and genetic factors influencing hemostasis in Yup’ik people and have important implications for treatment of this population with oral anticoagulants.

## Methods

### Study recruitment and sample collection

This study was conducted according to the guidelines in the Declaration of Helsinki and all procedures involving human study volunteers were approved by the Institutional Review Boards at the University of Alaska Fairbanks and the University of Washington, and by the Yukon–Kuskokwim Health Corporation Executive Board. Informed, written consent was obtained from all individuals prior to participation in the study. The procedure for obtaining consent was reviewed and approved by the YKHC Executive Board and IRB committees.

A cross-sectional, community-based, participatory research study of genetic and nutritional factors affecting blood coagulation in Yup’ik people living in the Yukon–Kuskokwim region of Southwestern Alaska was designed as a collaboration between the Northwest-Alaska Pharmacogenetic Research Network and the Center for Alaska Native Health Research (CANHR)[18]. In total, 733 study participants were recruited across ten communities between October 2010 and November 2013. Four communities were considered inland (greater than 5 miles away from the Bering Sea coast) and six were defined as coastal (within 5 miles of the Bering Sea coast). Participants were excluded from analysis if they self-reported their ethnicity as non-Alaska Native or ‘other’. Demographic data on age, sex, and community location were recorded. Blood was collected into 10 ml K2-EDTA, and 2.7 ml Na Citrate (BD Vacutainer^®^) tubes, and processed at the site of collection using a portable centrifuge. Samples were centrifuged for 15 minutes at 2500 rpm. Plasma, serum, and RBCs were isolated and stored in aliquots at -15°C in a portable freezer. Lymphocytes were isolated and stored in Puregene Cell Lysis Solution until DNA purification using the Gentra Puregene kit (Qiagen, Valencia, California, USA). All study participants fasted for 12 hours prior to blood draws and all samples were shipped to the University of Alaska Fairbanks within seven days and stored at -80°C prior to further analyses.

Samples for platelet function, clotting factor activity, and coagulation status measurements were sent to the University of Washington Medicinal Chemistry Department and subsequently transferred to Laboratory Medicine for these analyses. DNA samples were sent to the University of Washington Center for Ecogenetics and Environmental Health Functional Genomics and Proteomics Core lab for genotyping.

### Nitrogen isotope ratio analysis

The nitrogen isotope ratios of RBC samples were analyzed at the Alaska Stable Isotope Facility at the University of Alaska Fairbanks (UAF) by continuous-flow isotope ratio mass spectrometry as previously described by O’Brien et al. [17]. Nitrogen isotope ratios are represented as delta values, which give ‘permil (‰)’ abundance of heavy isotope relative to international standards: [δ^15^N = (R_sample_—R_standard_)/R_standard_] × 1000‰, where R is the ratio of heavy to light isotope, and the standard is atmospheric nitrogen (^15^N/^14^N atm-N = 0.0036765). Multiple peptone working standards (δ^15^N = 7.0, n = 128) were analyzed concurrently to assess analytical accuracy and precision, measured as the standard deviation of these analyses. Accuracy was within 0.1‰ and precision was within 0.2‰ [19]. The relationship between EPA and δ^15^N as previously reported was positive and linear (EPA = 1.04(δ^15^N)– 0.67, R^2^ = 0.70, p<0.0001), while that between DHA and δ^15^N was exponential non-linear and fitted as y = a (1-e^-r x^) (p<0.0001) [17].

### Clinical laboratory measurements

We included several coagulation indices to assess hemostasis in this study. All coagulation assays were performed at the University of Washington Department of Laboratory Medicine. Because samples were collected in remote regions where clinical laboratories are unavailable, platelet aggregation tests that require analysis within a few hours after collection were not feasible. Therefore, we used a surrogate marker for platelet activation, soluble P-selectin (sP-selectin), to assess the relationship between ω3 PUFA intake and platelet status. A commercially available sP-selectin ELISA kit (R&D systems), which contained microplates pre-coated with monoclonal antibodies specific for sP-selectin was used according to the manufacturer’s directions. Prothrombin time (PT) and partial thromboplastin time (PTT) was measured using a STA-Compact coagulation analyzer (Diagnostica Stagl). Clotting factors II and V were measured as percent activities, determined from a standard curve (log-based) prepared using dilutions of Unicalibrator against a normal plasma pool (Diagnostica Stago) containing known levels of each clotting factor. PIVKA-II was measured using a commercially available kit from Diagnostica Stago (Asserachrom PIVKA-II) with a limit of quantitation of 2.0 ng/mL. Fibrinogen concentration, quantitatively assessed by the Clauss clotting method, was used to determine whether clots found in samples would affect coagulation assays. Hemolyzed samples were excluded from sP-selectin and PIVKA-II analysis. Samples containing clots were excluded if fibrinogen concentrations were <150 ng/mL.

### Vitamin K analysis in plasma

#### Reagents and standards

Vitamin (VK1) was obtained from Supelco and deuterated vitamin K1 (VK1-d7) from Aldrich. Vitamin K standards were prepared in methanol and concentrations confirmed using a molar extinction coefficient of 19000 M^-1^ at 248 nm [20, 21].

#### Plasma sample preparation for vitamin K analysis

All handling procedures for vitamin K analysis were performed under reduced (yellow) light. Plasma samples (0.5 ml) were prepared in duplicate prior to LC-MS analysis and extracted using minor modifications to the method of Paroni et al [22], after addition of 4.0 ng/ml of VK1-d7. Ethanol (2.0 mL) was added, and vigorously mixed. Samples were centrifuged at 3,000 rpm for 5 minutes, the supernatant transferred into a separate tube and then applied to 1.0 mL Oasis HLB Polymeric SPE columns. The column cartridges were initially conditioned with 1.0 mL of methanol and 1.0 mL of water. Samples were loaded onto the columns and then washed with 1.0 mL of water, 1.0 mL of 50:50 (v/v) water/methanol, and 1.0 mL 20:80 (v/v) water/methanol. The columns were dried in air for 1 minute and finally eluted with 1.0 mL of acetonitrile/isopropanol/dichloromethane (70:10:20). The eluate was evaporated under nitrogen and the residue dissolved in 100 *μ*L of methanol for analysis of 15 *μ*L aliquots that were automatically injected onto the LC/MS system for determination of plasma vitamin K1 concentrations.

#### LC-MS conditions for plasma vitamin K analysis

Plasma vitamin K1 was analyzed by LC-MS with modifications to the method of Fu et al. [23]. The assay was established on a Waters TQ-S mass spectrometer connected to a Waters Acquity I-Class UPLC system with an APCI source operating in negative mode. The APCI probe temperature was set to 650°C. The corona voltage was set to 30 μA and the desolvation gas flow was 500 L/hr. The temperature-controlled UPLC column compartment was set to 50°C. Chromatographic separation of analytes was achieved on a Waters Acquity UPLC BEH phenyl 1.7 *μ*m, 2.1 x 50 mm column with a Phenomenex SecurityGuard Cartridge System equipped with a 4.0 x 2.0 mm C8 SecurityGuard cartridge. The mobile phase consisted of solvent A (water) and solvent B (0.05% formic acid in methanol). A linear gradient was run as follows: 85% solvent B at 0 minutes to 100% solvent B at 3 minutes with a flow rate of 0.35 mL/min. Elution continued with Solvent B at 100% for 3 more minutes before re-equilibration back to 85% solvent B over a final 2 minutes. The cycle was complete at 8 minutes. Under these conditions vitamin K1 eluted at 3.5 min. Optimized cone and collision voltage conditions for the analyte were 58V and 30V, respectively. The linearity of response was tested by spiking the analyte into 0.5 mL of FBS in concentrations that ranged from 0.10 to 2.00 ng/mL. Linear regression was used to compare measurements obtained by LC-MS to expected concentrations of calibration standards. Standard curves were linear with R^2^ values of >0.99. The limit of quantitation was 0.20 ng/mL. Plasma samples were excluded from data analysis if the variation in measured vitamin K between duplicates was greater than 20%.

### Genotyping of CYP4F2, VKORC1 and GGCX

SNPs for CYP4F2*3 (rs2108622), *GGCX R325Q* (rs 699664), and *VKORC1 1173 G>A* (rs9934438) were analyzed using TaqMan SNP Genotyping Assays (Applied Biosystems, Inc.) on 96.96 Dynamic Genotyping Arrays (Fluidigm). Dynamic Arrays were primed and loaded on the Fluidigm HX and thermo-cycled on the Fluidigm FC1 controller. End-point fluorescence was read on a BioMark^™^ Real-Time PCR System (Fluidigm) and analyzed using SNP Genotyping Analysis software (Fluidigm). Samples with call rates <95% were excluded from analysis. A subset of genotypes samples were selected for DNA sequencing with >99.5% concordance between the two methods. Methods and allele frequencies for each of these variants are detailed in a recent paper [16].

### Statistical analysis

Statistical analyses were performed using STATA (version 11.0 SE) and a P-value ≤0.05 was considered significant for all tests. In total, samples from 733 study participants were evaluated for an association with measures of coagulation (where available). Of these, 682 had measureable PIVKA-II data free from interferences and complete information on genotype data for CYP4F2*3, *GGCX R325Q* and *VKORC1*-*1173 T>C* (excluding no calls). Due to limited availability of plasma, a subset of 185 plasma samples that had complete genotype information was analyzed for vitamin K content. We evaluated the relationship between the δ^15^N value and measures of coagulation (sP-selectin, activities of clotting factors II and V, fibrinogen, PT/INR, and PTT) using univariate and multivariate linear regression models. Measures of coagulation were natural log-transformed prior to statistical analysis. δ^15^N was treated as an independent variable and measures of coagulation were treated as dependent variables. Study participant age and sex were included as covariates in regression analyses. A two-sample t-test was used to compare δ^15^N values between groups and a community’s geographical status (coastal or inland). Differences in measures of coagulation were also compared between geographical status groups using the two-sample t-test.

Subject samples were coded 0, 1, or 2 for alleles of interest. To evaluate the association between plasma vitamin K levels and *CYP4F2*, *VKORC1*, and *GGCX* genotypes, a multivariate gene-dosing regression model was used for statistical analysis that included age, sex, and geographical status (from which the plasma samples were obtained) as covariates. Plasma vitamin K levels were natural-log transformed to improve normality prior to statistical analysis. Genotype categories were classified for all genes as having zero, one, or two copies of the variant allele.

A logistic regression model was used determine the association between long-term vitamin K status and *CYP4F2*, *VKORC1* and *GGCX* genotypes. The outcome was dichotomous and categorized as either PIVKA-II ≥2.0 ng/mL (low vitamin K status) or PIVKA-II <2.0 ng/mL (normal vitamin K status). The predictor variables of interest were genotype (0, 1, or 2 copies of variant allele), age (continuous), sex (binary) and whether the study community was located in a coastal or inland region (binary). The OR and confidence interval (CI) of 95% were reported. The overall fit of the model to the data was evaluated using the Likelihood Ratio Test (χ^2^) and Goodness-of-Fit Test (Pearson χ^2^). A two-tailed p-value ≤0.05 was considered significant for all statistical tests. Only study participants with complete information on PIVKA-II (or plasma vitamin K) and genotype data (*CYP4F2*, *VKORC1* and *GGCX)* were included for statistical analysis.

## Results

### Study participant demographics

The demographic characteristics of Yup’ik study participants and descriptive statistics for δ^15^N value are shown in Table 1. The mean age of all study participants was 37 years and 47% were female. The mean δ^15^N value across all participants was 8.7 ± 1.3‰, and the range was 6.1 to 14.5‰.

**Table 1:** Demographic characteristics of the Yup’ik study participants and descriptive statistics for the δ^15^N value.

	All	Males	Females
Sample size	733	388	345
Age (mean ± S.D.)	36.8 ± 18.1	34.6 ± 17.4	39.3 ± 18.6
Range of ages	14–93	14–85	14–93
δ^15^N values (mean ± S.D)	8.7 ± 1.3	8.4 ± 1.1	**9.1 ± 1.4**
Range of δ^15^N values	6.10–14.5	6.10–13.2	6.59–14.51
	**Coastal Communities**	**Inland Communities**	
Number of males	204	184	
Number of females	182	163	
All δ^15^N values (mean ± S.D)	9.1 ± 1.5	**8.4 ± 0.9**	
Male δ^15^N values (mean ± S.D)	8.6 ± 1.3	**8.1 ± 0.8**	
Female δ^15^N values (mean ± S.D)	9.5 ± 1.5	**8.7 ± 1.0**	

δ^15^N is a surrogate for ω3 PUFA intake.

Bolding denotes a significant difference (P<0.05) in δ^15^N values between males and females and between coastal and inland communities.

**Fig 2:**
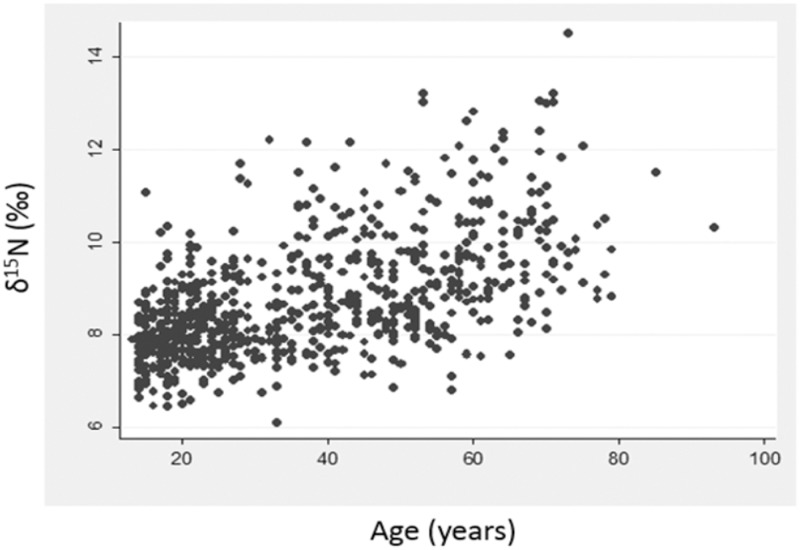
Plot of δ^15^N values in the study sample and association with age. (N = 733, P<0.001, R^2^ = 0.330). Higher δ^15^N values indicate increased intake of ω3 PUFAs.

Participant age correlated positively with δ^15^N values (Fig 2), females had higher mean δ^15^N values than males (Table 1, Fig A in S1 File) and coastal communities exhibited higher mean δ^15^N values compared to inland communities (Table 1, Fig B in S1 File). These data are in accordance with previous findings on dietary trends and traditional food intake in the same Yup’ik study population [19].

### Association of δ^15^N values with coagulation parameters

Results of the regression analysis with coagulation variables before and after adjustment for age, sex and geographical status are shown in Table 2, with detailed information on these variables before and after natural log transformation provided in Table A, S1 File.

**Table 2:** Association of δ^15^N values with coagulation variables using multivariate regression analysis.

		Unadjusted			Adjusted		
Variable	N	β Coefficient (95% CI)	P-value	R^2^	β Coefficient (95% CI)	P-value	R^2^
sP-selectin	716	-0.051 (-0.070, -0.033)	**<0.001**	0.039	-0.069 (-0.093, -0.044)	**<0.001**	0.107
Clotting factor II	708	0.007 (-0.004, 0.018)	0.196	0.002	0.002 (-0.012, 0.017)	0.776	0.019
Clotting factor V	705	0.035 (1.02E-4, 0.069)	**0.049**	0.006	0.007 (-0.040, 0.054)	0.759	0.013
Fibrinogen	358	0.038 (0.013, 0.063)	**0.003**	0.025	-1.01E-4 (-0.035, 0.034)	0.995	0.067
PT	722	-0.005 (-0.012, 0.001)	0.122	0.003	0.002 (-0.007, 0.012)	0.603	0.014
INR	721	-0.005 (-0.014, 0.004)	0.273	0.002	0.005 (-0.007, 0.017)	0.455	0.015
PTT	449	0.006 (-0.008, 0.021)	0.390	0.002	0.007 (-0.012, 0.025)	0.481	0.088

The significance level was set at P<0.05, denoted in bold.

Statistical estimates are presented before and after adjustment for age, sex and geographical status.

**Fig 3:**
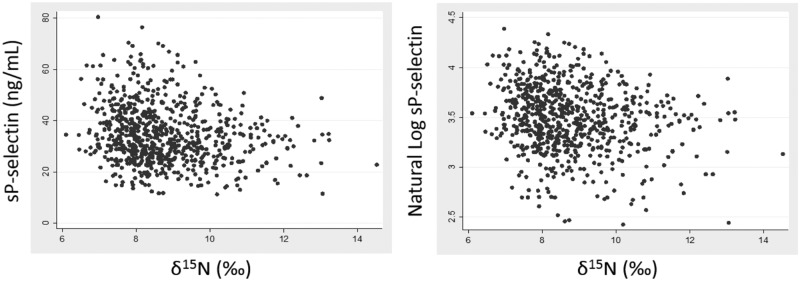
Relationship between δ^15^N and sP-selectin, before and after natural log transformation. A statistically significant, negative association of the log-transformed data was obtained before (P<0.001, R^2^ = 0.039) and after adjustment for age, sex and geographical status (P<0.001, R^2^ = 0.107).

No significant associations were observed between δ^15^N values and factor II, factor V, fibrinogen, PT, INR or PTT. However, sP-selectin levels varied inversely with δ^15^N values, before and after adjustment (Table 2, Fig 3), demonstrating that higher ω3 PUFA consumption was associated with lower platelet activity

### Vitamin K levels in plasma and relationship to genotypes

**Fig 4:**
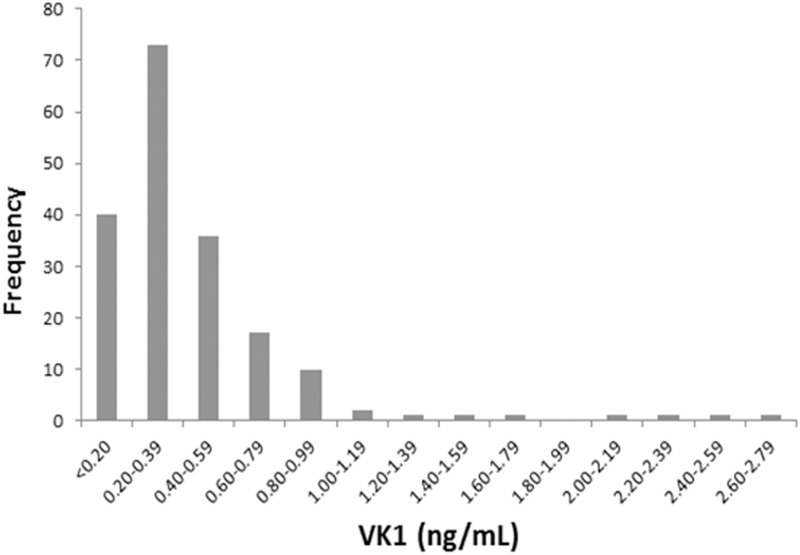
Frequency distribution of plasma VK1 concentration (N = 185). 20.5% of samples had VK1 concentrations of <0.20 ng/ml.

Plasma VK1 concentrations ranged from <0.20–2.80 ng/ml (Fig 4). The mean ± standard deviation (S.D.) of VK1 in plasma of all Yup’ik samples analyzed (N = 185) was 0.45 ± 0.39 ng/mL (Table 3). A statistically significant association was observed between plasma VK1 concentration and CYP4F2*3, but not with either *GGCX R325Q* or *VKORC1 1173 A>G* (Table 4). When VK1 concentrations were stratified by CYP4F2*3 genotype, the mean and median plasma concentration were highest for CYP4F2 *3/*3 compared to the *1/*3 or *1/*1 genotypes (Table 3). Furthermore, these differences relative to the *1/*1 genotype group were significant for *3/*3 (P = 0.005), but not for *1/*3 (P = 0.685) (Fig C, S1 File).

**Table 3:** Relationship between plasma VK1 and *CYP4F2*, *GGCX*, and *VKORC1* genotypes.

Group	N	VK1 (ng/mL)	
		Mean ± S.D.	Median (IQR)
**All**	185	0.45 ± 0.39	0.33 (0.32)
CYP4F2*3			
*1/*1	49	0.39 ± 0.34	0.30 (0.34)
*1/*3	87	0.38 ± 0.25	0.30 (0.27)
*3/*3	49	0.61 ± 0.57	0.42 (0.43)
***GGCX R325Q***			
CC	42	0.42 ± 0.30	0.31 (0.30)
CT	98	0.50 ± 0.47	0.34 (0.39)
TT	45	0.35 ± 0.23	0.27 (0.28)
***VKORC1 1173 A>G***			
AA	128	0.44 ± 0.34	0.35 (0.35)
AG	48	0.40 ± 0.35	0.30 (0.28)
GG	9	0.75 ± 0.88	0.32 (0.54)

**Table 4:** Association of plasma vitamin K with *CYP4F2*, *GGCX and VKORC1* genotypes.

		Unadjusted			Adjusted		
Genotype	N	β Coefficient (95% CI)	P-value	R^2^	β Coefficient (95% CI)	P-value	R^2^
CYP4F2*3	185	0.205 (0.073, 0.336)	**0.002**	0.049	0.191 (0.058, 0.324)	**0.005**	0.067
***GGCX R325Q***		-0.083 (-0.225, 0.060)	0.253	0.007	-0.079 (-0.221, 0.063)	0.276	0.032
***VKORC1 1173 A>G***		-0.004 (-0.176, 0.167)	0.960	<0.001	-0.017 (-0.189, 0.154)	0.841	0.026

The significance level was set at P<0.05, denoted in bold.

Statistical estimates are presented before (upper) and after (lower) adjustment for age, sex and geographical status.

### PIVKA-II status and relationship to genotypes

**Fig 5:**
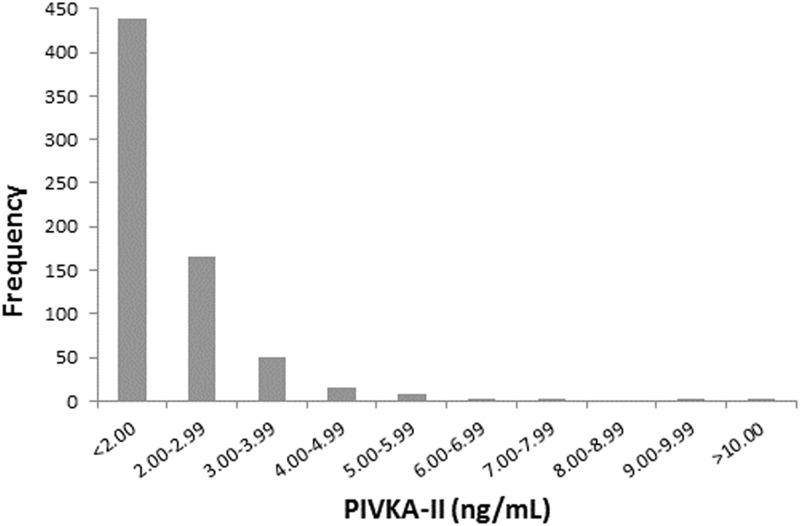
Frequency distribution of plasma PIVKA-II (N = 682). 36.2% of samples had PIVKA-II concentrations equal to or above 2.0 ng/mL. Two values not included were outliers with plasma levels of 1618 and 1708 ng/mL.

Among study participants in which PIVKA-II and genotype data were complete, 247 (36.2%) had values ≥ 2.00 ng/mL; two samples exhibited levels >1000 ng/ml and were excluded from analysis (Fig 5). Similar to the data obtained for plasma VK1, PIVKA-II concentrations varied only with CYP4F2*3 genotype (Table 5). Table B, S1 File provides demographic information for this group. There was no significant difference in age between study participants who had low or normal PIVKA-II levels (two-sided t-test, p = 0.256). No significant difference in PIVKA-II status was observed between coastal and inland communities (Pearson-Chi^2^, p = 0.181). However, a significantly higher proportion of males had an elevated PIVKA-II level compared to females (Pearson-Chi^2^, p = 0.008).

**Table 5:** Effect of *CYP4F2*, *GGCX* and *VKORC1* genotype on plasma PIVKA-II.

Group	Sample Size	% with PIVKA-II≥2.0 ng/mL
All	682	36.2
CYP4F2*3		
*1/*1	185	42.7
*1/*3	329	35.3
*3/*3	168	31.0
*GGCX R325Q*		
CC	164	37.2
CT	354	36.5
TT	164	33.1
*VKORC1 1173 A>G*		
AA	433	37.2
AG	199	35.7
GG	50	30.0

Data are from samples that had complete information on genotype status (excluding no calls) and PIVKA-II analysis that passed quality control standards.

The association analysis of *CYP4F2*, *GGCX*, and *VKORC1* genotypes from the multivariate logistic regression is presented in Table 6. The P-value was <0.05 from the Likelihood Ratio Test for all genotypes and indicated that the model is statistically significant. The P-value was >0.05 from the Goodness-of-Fit Test for all genotypes, which indicated that the models fit reasonably well to the data. The CYP4F2*3 allele was found to associate with lower odds (OR = 0.74) of low vitamin K status. When homozygous variants and heterozygotes were individually compared to the homozygous reference allele group, the association was stronger for *3/*3 (OR = 0.60) than for the *1/*3 allele group (OR = 0.74). *GGCX R325Q* and *VKORC1 1173 A>G* genotypes were not significantly associated with PIVKA-II. Interestingly, sex was consistently associated with PIVKA-II after controlling for *GGCX R325Q* and *VKORC1 1173 A>G* genotypes and the other covariates, similar to what was observed in the univariate analysis.

**Table 6:** Multivariate association analysis of PIVKA-II status and CYP4F2*3, *GGCX R325Q*, and *VKORC1-1173 A>G* genotypes.

**Genotype** (Covariate)	**Odds Ratio**	**P-value**	**95% CI**
CYP4F2*3	0.741	**0.009**	0.591–0.929
(Age)	0.996	0.446	0.988–1.01
(Sex)	0.650	**0.008**	0.472–0.895
(Geographic Status)	0.735	0.061	0.532–1.01
**Genotype** (Covariate)	**Odds Ratio**	**P-value**	**95% CI**
*GGCX R325Q*	0.916	0.449	0.729–1.15
(Age)	0.996	0.346	0.987–1.00
(Sex)	0.654	**0.009**	0.475–0.900
(Geographic Status)	0.798	0.161	0.582–1.09
**Genotype** (Covariate)	**Odds Ratio**	**P-value**	**95% CI**
*VKORC1 1173 A>G*	0.862	0.258	0.667–1.13
(Age)	0.996	0.350	0.987–1.00
(Sex)	0.659	**0.010**	0.479–0.906
(Geographic Status)	0.777	0.120	0.565–1.07

Odd ratios and 95% confidence intervals (CI) for plasma PIVKA-II ≥2.0 ng/ml (PIVKA-II status)) compared to participants with PIVKA-II levels <2.0 ng/ml are shown for multivariate logistic regression analysis of CYP4F2*3, *GGCX R325Q*, and *VKORC1 1173 A>G* genotypes with age, sex, and geographic location as covariates.

Odds ratios that are significantly different from 1 at the 0.05 significance level have P-values shown in bold.

The reference group for sex and geographical status was male and inland, respectively.

The reference group for the three genotypes were CYP4F2*1, *GGCX R325* and *VKORC1 1173A*. Age was a continuous variable.

## Discussion

In the first part of this study, we evaluated whether high dietary ω3 PUFA intake in Yup’ik people, as indicated by larger δ^15^N values, might contribute to bleeding diathesis. We observed that δ^15^N was inversely associated with the platelet biomarker, sP-selectin. The inverse association between sP-selectin and the δ^15^N value supports the contention that a platelet inhibition effect is promoted by higher consumption of ω3 PUFAs. Some studies have reported that sP-selectin decreases only after high doses of ω3 PUFA supplements are consumed [24, 25]. Our data suggest that increasing ω3 PUFA intake from a natural diet is inversely associated with platelet activity, although a direct analysis of platelet aggregation and function in a more controlled setting is warranted. In contrast, we observed no significant association between factor II, factor V or fibrinogen and δ^15^N. Furthermore, we did not find evidence that higher δ^15^N values prolonged PT, INR, or PTT. Therefore, the most likely mechanism for a bleeding diathesis in our study population is through the selective inhibitory effects of ω3 PUFAs on platelet activity. It would be of interest to determine if this association is any different for patients on anticoagulant or antithrombotic therapy.

A second focus of our study was to evaluate vitamin K status in this population and determine whether this was associated with common vitamin K cycle polymorphisms; CYP4F2*3, *GGCX R325Q*, and *VKORC1 1173 G>A* genotypes. Notably, a little over one-third of our study population exhibited high PIVKA-II levels—indicative of low vitamin K status—however, only CYP4F2*3 genotype was a statistically significant factor affecting both PIVKA-II levels and plasma vitamin K. CYP4F2 is a ω-hydroxylase for VK1 and menaquinone-4 and variation in the *CYP4F2* gene, specifically CYP4F2*3, is associated, in most populations studied, with a higher warfarin dose requirement of approximately 1 mg/day [26, 27]. The finding that CYP4F2*3 genotype positively associates with plasma VK1 concentrations and that these levels are increased in study participants carrying the *3/*3 genotype relative to *1/*3 and *1/*1 are consistent with the idea that CYP4F2*3 may help conserve vitamin K in the liver by slowing its hepatic metabolism [27]. The CYP4F2*3 allele results in a lower hepatic concentration of the variant enzyme, relative to wild-type, which is the basis for its reduced catalytic function [27]. Two other recent genetic studies help substantiate a role for CYP4F2 in vitamin K disposition *in vivo* [28, 29]. CYP4F2*3 genotype also influences α-tocopherol (vitamin E) levels in plasma [30]. Therefore, in the aggregate, these data are suggestive of a broad role for CYP4F2*3 genotype in the disposition of some fat-soluble vitamins.

The potential of the CYP4F2*3 allele to maintain higher levels of VK1 in plasma may have an impact on chronic hepatic vitamin K status. Both plasma vitamin K and PIKVA-II are influenced by dietary vitamin K intake, but PIVKA-II is a more commonly measured biomarker of vitamin K status due to its longer half-life relative to serum VK1 [31–34]. Data from our logistic regression model suggest that the CYP4F2*3 allele reduces the likelihood of having low vitamin K status, and this effect was most evident in the *3/*3 homozygotes. Similar to our results for plasma VK1, PIVKA-II levels were not influenced by *GGCX R325Q* or *VKORC1 1173 G>A* polymorphisms. However, these latter findings for *VKORC1* may reflect the low sample numbers in the variant genotype groups, where we calculated power to be only 17% to detect a significant odds ratio. Future studies investigating the relationship between *VKORC1 1173* G>A and vitamin K status will require a much larger number of Yup’ik individuals with the minor GG genotype to have adequate power to detect an association.

Another limitation of this study is that platelet activation and blood coagulation assays could not be performed at the time of sample collection due to the unavailability of clinical laboratories in the rural Alaska communities where study participants were recruited. However, this is an unavoidable aspect of research ‘in the field’ and because all samples were processed in an identical manner prior to analysis, inter-sample variability should have been minimized. It should also be noted that sP-selectin and the δ^15^N value are biomarkers for platelet activity and EPA/DHA levels, respectively, and that any associations obtained using these parameters are indirectly inferred. Nonetheless, both biomarkers have been validated by other researchers, so we have confidence in the robustness of the associations observed in this study. Finally, because our study design was cross-sectional, we were not able to establish causality.

In summary, we found that δ^15^N values are highly correlated with the platelet biomarker sP-selectin in Yup’ik people living in the Yukon-Kuskokwim delta. EPA/DHA derived from a traditional marine diet may blunt platelet activation and reduce the risk of cardiovascular and/or inflammatory disease states, which has been reported for other studies [35–37]. Our data also indicate that the CYP4F2*3 polymorphism is associated with both acute and long-term variation in vitamin K status. Specifically, high frequency of the CYP4F2*3 allele observed in Yup’ik people is consistent with the hypothesis that preservation of this low activity, vitamin K hydroxylase allele could be a mechanism to safeguard against low vitamin K status. This could reduce the risk of a hypocoagulative state in an environment with less dietary availability of vitamin K (i.e. limited commercial availability of green vegetables). While this may play a less significant role today, given a dietary shift to include more commercial market foods, our findings are likely still to be important pharmacogenetic considerations when prescribing anticoagulant medications and treating chronic disease in this population of Alaska Native peoples.

## Supporting information

S1 File **Table A, S1 file.** Characteristics of δ^15^N values and coagulation parameters from samples analyzed in this study.**Table B, S1 file.** Demographics for the participant group that provided samples for PIVKA-II analysis.**Fig A, S1 file.** Sex differences in δ^15^N values among Yup’ik participants.**Fig B. S1 file.** Distribution of δ^15^N values from study participants stratified by; A) coastal and B) inland communities.**Fig C, S1 file.** Effect of *CYP4F2* genotype on plasma vitamin K levels.
(PDF)
