# Supplementary material for: Dietary and genetic influences on hemostasis in a Yup’ik Alaska Native population
Source: PLoS One. 2017 Apr 4;12(4):e0173616. doi: 10.1371/journal.pone.0173616 (PMC5380313; doi:10.1371/journal.pone.0173616)

Table A, S1 file

Characteristics of $\delta^{15}N$ values and coagulation parameters from samples analyzed in this study.

Variables were natural-log transformed to improve the normality of the frequency distributions. Skewness and kurtosis values are given for data before and after natural-log transformation of variables. For simplicity, only natural-log transformed dependent (coagulation) variables were used in regression analysis. $\delta^{15}N$ was used as the independent variable and was not transformed. A variable with symmetrical distribution has a skewness of zero. The kurtosis for a standard normal distribution is three.

	**Untransformed Values**				
Variable	Mean ± S.D.	Median (IQR)	Range	Skewness	Kurtosis
$\delta^{15}N$ value (‰)	8.7 ± 1.3	8.4 (1.58)	6.1-14.5	1.06	4.26
sP-selectin (ng/mL)	34.6 ± 11.4	33.2 (14.3)	11.3-80.4	0.72	3.62
Clotting factor II (%)	107.4 ± 20.1	103.0 (22.0)	35.0-180.0	0.80	4.01
Clotting factor V (%)	81.6 ± 32.0	87.0 (38.0)	3.5-211.0	-0.47	3.05
Fibrinogen (ng/mL)	345 ± 106	333.0 (124.0)	142-842	0.70	4.37
PT (sec)	12.7 ± 1.61	12.7 (1.40)	8.0-25.0	1.91	14.2
INR	1.01 ± 0.17	1.00 (0.200)	0.6-2.5	2.47	19.3
PTT (sec)	33.8 ± 10.4	32.0 (6.00)	14-199	9.88	148.1
	**Natural Log-Transformed Values**				
Variable	Mean ± S.D.	Median (IQR)	Range	Skewness	Kurtosis
$\delta^{15}N$ value (‰)	2.16 ± 0.14	2.13 (0.18)	1.81-2.67	0.66	3.28
sP-selectin (ng/mL)	3.49 ± 0.33	3.50 (0.43)	2.43-4.39	-0.27	3.19
Clotting factor II (%)	4.66 ± 0.18	4.63 (0.21)	3.55-5.19	-0.11	5.70
Clotting factor V (%)	4.27 ± 0.60	4.47 (0.46)	1.25-5.35	-1.87	6.53
Fibrinogen (ng/mL)	5.79 ± 0.32	5.81 (0.37)	4.84-6.74	-0.40	3.44
PT (sec)	2.54 ± 0.12	2.54 (0.11)	2.19-3.46	0.64	7.31
INR	$-4.02 \times 10^{-4} \pm 0.15$	0.00 (0.20)	-0.51-0.92	0.69	7.22
PTT (sec)	3.50 ± 0.20	3.47 (0.18)	2.64-5.29	2.23	21.2

Table B, S1 file

Demographics for the participant group that provided samples for PIVKA-II analysis.

Group	Sample Size	Age (years)	Age Range	% Female	% Coastal
All	682	36.2 ± 17.8	14-79	47.2	52.8
*CYP4F2*3*					
*1/*1	185	33.6 ± 17.6	14-79	48.6	63.2
*1/*3	329	37.1 ± 17.8	14-77	47.1	54.7
*3/*3	168	37.6 ± 17.8	14-79	45.8	37.5
GGCX R325Q					
CC	164	35.3 ± 17.2	14-77	53.7	53.7
CT	354	36.7 ± 18.1	14-78	47.2	50.3
TT	164	36.2 ± 17.8	14-79	40.9	57.3
VKORC1 1173 A>G					
AA	433	35.9 ± 17.9	14-79	47.8	58.0
AG	199	36.8 ± 17.5	14-79	46.2	43.7
GG	50	37.4 ± 18.1	14-77	46.0	44.0

Fig A, S1 file

Sex differences in $\delta^{15}N$ values among Yup'ik participants.

Females had higher $\delta^{15}N$ values compared to males ($P<0.001$). Median (IQR) $\delta^{15}N$ for males was 8.2 (1.3) and for females was 8.8 (1.7).

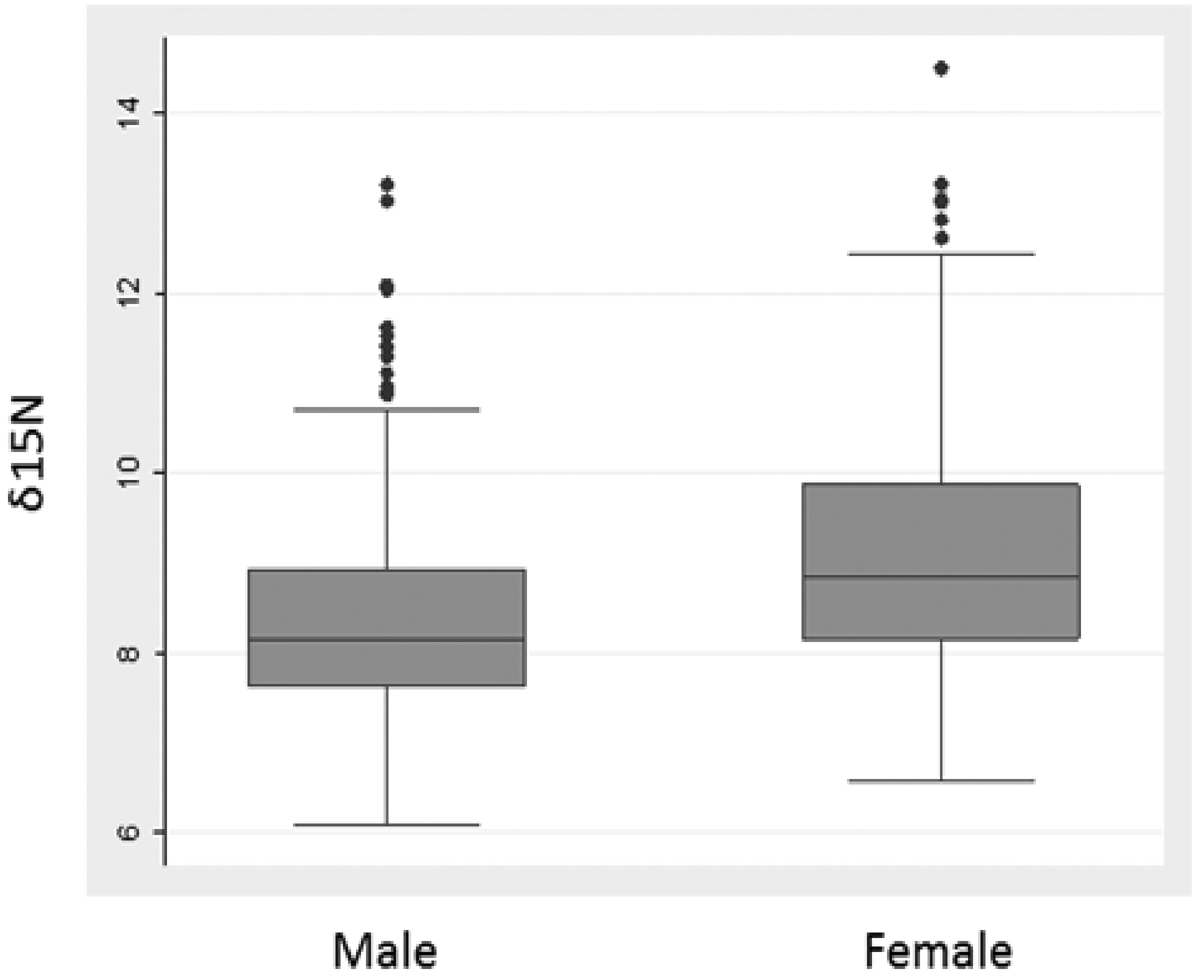

Fig B, S1 file

Distribution of δ^{15}N values from study participants stratified by; A) coastal and B) inland communities.

Coastal communities generally exhibited higher δ^{15}N values (mean ± S.D., 9.1 ± 1.5) than inland communities (8.4 ± 0.9); $P<0.001$.

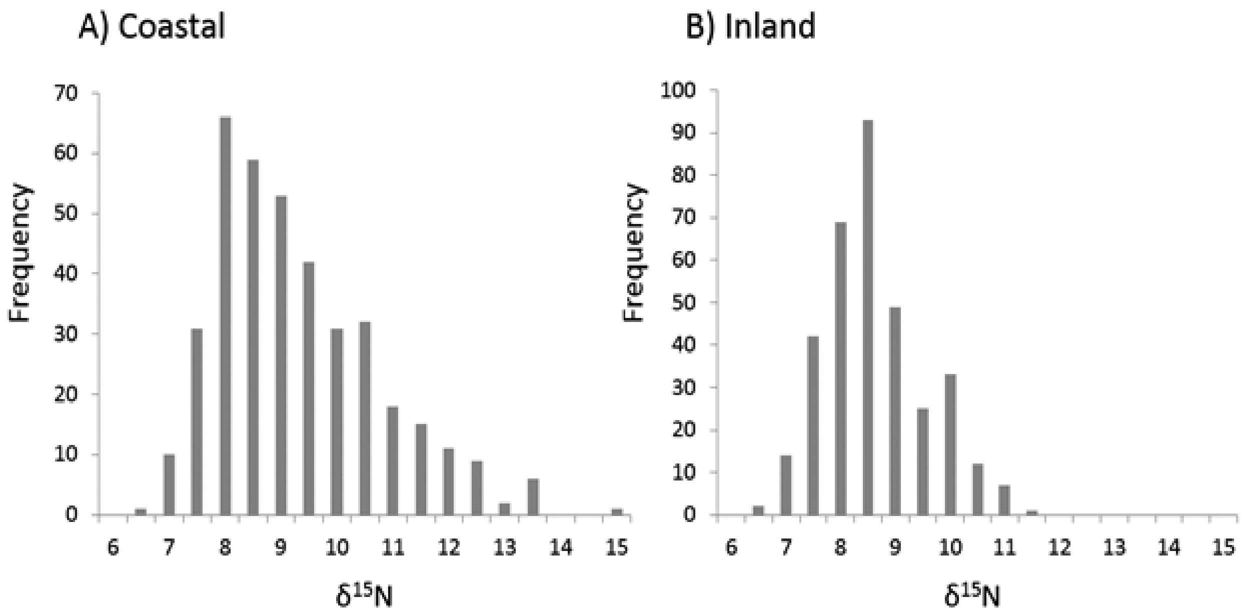

Fig C, S1 file

Effect of *CYP4F2* genotype on plasma vitamin K levels.

Box-and-whisker plots comparing medians and IQRs of plasma VK1 concentrations for *CYP4F2*3.* Statistical data are summarized in Table 3. Using a two-sided t-test for comparing natural-log transformed plasma VK1 values, P=0.005 for **3/*3* vs. **1/*1*, p=P.685 for **1/*3* vs. **1/*1*.

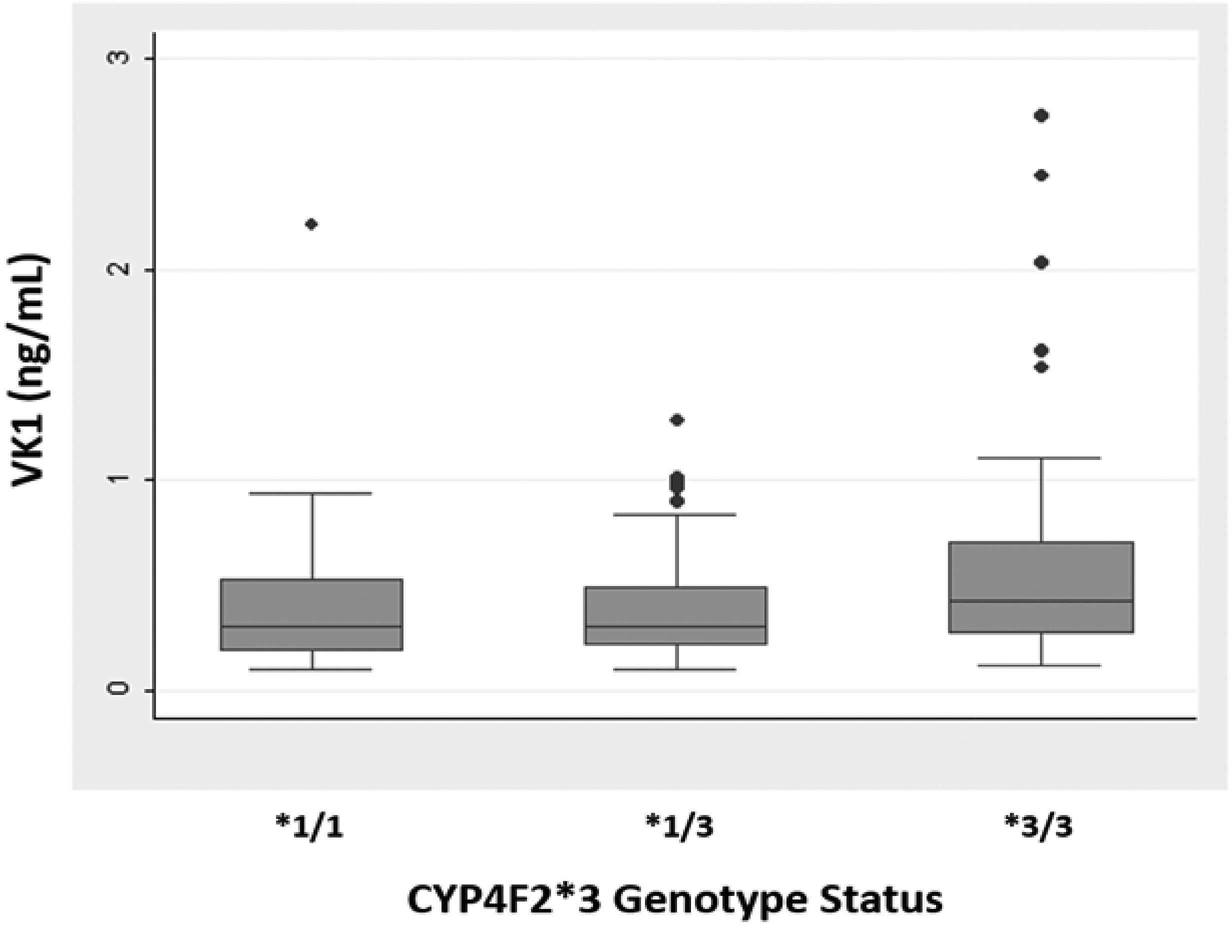

Supplement: S1 File — Table A, S1 file. Characteristics of δ15N values and coagulation parameters from samples analyzed in this study.Table B, S1 file. Demographics for the participant group that provided samples for PIVKA-II analysis.Fig A, S1 file. Sex differences in δ15N values among Yup’ik participants.Fig B. S1 file. Distribution of δ15N values from study participants stratified by; A) coastal and B) inland communities.Fig C, S1 file. Effect of CYP4F2 genotype on plasma vitamin K levels. (PDF) [file pone.0173616.s001.pdf]
